# Zebrafish as a multimodal platforms for anti-inflammatory phytomedicine discovery and translation

**DOI:** 10.3389/fphar.2026.1775366

**Published:** 2026-03-25

**Authors:** Xiaohui Tan, Huazhen Liu, Fang Liang, Ganlin Chen

**Affiliations:** 1 Guangxi Subtropical Crops Research Institute, Guangxi Academy of Agricultural Sciences, Nanning, China; 2 Guangxi Key Laboratory of Quality and Safety Control for Subtropical Fruits, Guangxi Subtropical Crops Research Institute, Nanning, China; 3 Key Laboratory of Quality and Safety Control for Subtropical Fruit and Vegetable, Ministry of Agriculture and Rural Affairs, Nanning, China; 4 Shanghai Institute of Traumatology and Orthopaedics, Ruijin Hospital, Shanghai Jiao Tong University School of Medicine, Shanghai, China; 5 College of Smart Agriculture, Yulin Normal University, Yulin, China

**Keywords:** anti-inflammatory, multimodal platforms, phytochemicals, translational strategies, zebrafish model

## Abstract

The clinical translation of plant-derived anti-inflammatory agents is hindered by the disconnect between high-throughput *in vitro* assays and low-throughput, costly mammalian models. This review posits the zebrafish (*Danio rerio*) not merely as an alternative model, but as a central integrating platform that bridge this translational chasm through multimodal technological convergence. We first synthesize how zebrafish inflammation models—established via chemical, physical, or infectious stimuli—enable rapid, whole-organism efficacy and safety profiling of phytochemicals. We then dissect the conserved molecuar circuitry (NF-κB, MAPK, Nrf2) through which standardized extracts and isolated compounds exert their anti-inflammatory effects, visualized dynamically in transgenic lines. Crucially, we move beyond descriptive cataloguing to critically evaluate the integration of zebrafish with microfluidics-enabled high-content screening, AI-driven phenotypic analytics, CRISPR/Cas9 gene editing, network pharmacology, and humanized xenograft models. We argue that this convergent multimodal strategy transforms zebrafish from a correlative screening tool into a predictive, mechanism-resolving, and patient-relevant avatar system. We conclude that zebrafish-centric platforms furnish a robust, mechanism-driven, and scalable framework to accelerate the discovery, mechanistic elucidation, and rational preclinical development of safer, multi-target phytotherapeutics, thereby catalyzing the evolution of plant-based medicine from empirical discovery to precision anti-inflammatory therapy guided by a systems-level, mechanism-driven paradigm. This framework positions zebrafish-centered platforms as a cornerstone of next-generation anti-inflammatory precision medicine.

## 1 Introduction

Inflammation is a fundamental host defense response, essential for protecting against pathogen invasion, clearing damaged cells, and promoting tissue repair (Serhan and Savill, 2005; McInnes and Schett, 2017; Kondylis and Pasparakis, 2019). However, when dysregulated, excessive or persistent inflammation becomes pathogenic and drives tissue destruction, organ dysfunction, and the onset or progression of numerous chronic diseases, including cardiovascular disorders, diabetes, neurodegenerative conditions, and malignancies (Cross et al., 2014; Abraham and Cho, 2009; Apostolopoulou et al., 2025). Whereas acute inflammation confers short-term benefits by restoring homeostasis, chronic inflammation contributes to disease progression through mechanisms such as DNA damage, aberrant apoptosis, and fibrotic remodeling (Kotas and Medzhitov, 2015). Consequently, the precise modulation of inflammatory responses has emerged as a central goal of both basic and translational research. Conventional anti-inflammatory agents, including non-steroidal anti-inflammatory drugs and glucocorticoids, remain the clinical mainstay for rapid suppression of inflammation and pain. Yet, long-term or high-dose administration is frequently limited by serious adverse effects—gastrointestinal bleeding, cardiovascular risk, osteoporosis, and immunosuppression among them (Wongrakpanich et al., 2018; Griggs et al., 2013). These limitations underscore an urgent need for innovative therapeutic strategies that are not only safer but also mechanistically more selective.

Phytochemicals, bioactive compounds derived from natural products, have attracted growing attention. Owing to their structural diversity and multi-target activities, they regulate interconnected pathways involved in inflammation, oxidative stress, and immune responses (Khan et al., 2020). Representative examples include flavonoids, polyphenols, and alkaloids, which exert anti-inflammatory and tissue-protective effects via pathways such as NF-κB (Nuclear Factor Kappa-Light-Chain-Enhancer of Activated B Cells), MAPK (Mitogen-Activated Protein Kinase), and the NLRP3 (NLR Family Pyrin Domain Containing 3) inflammasome (Zhao et al., 2025; Lee et al., 2024; Dong et al., 2025). Notably, curcumin encapsulated in ferritin nanoparticles markedly reduced neutrophil infiltration and pro-inflammatory cytokine expression in zebrafish neuroinflammation models, while flavonoids from *Artemisia vestita* demonstrated dual inhibition of COX-2 (Cyclooxygenase-2) and 5-LOX (5-Lipoxygenase) (Chen et al., 2025a; Zhou et al., 2023). Compared with synthetic drugs, plant-derived compounds typically display lower intrinsic toxicity and achieve broad therapeutic effects through synergistic, multi-pathway regulation—that positions them as valuable lead structures for drug discovery (Atanasov et al., 2021; Mittal and Sharma, 2024). Nevertheless, their remarkable chemical diversity and complex pharmacology pose major obstacles to clinical translation, particularly in early-stage screening, activity validation, and mechanistic dissection.

Traditional *in vitro* assays often fail to recapitulate the complexity of *in vivo* inflammatory processes, whereas mammalian models are costly, time-consuming, and poorly suited to large-scale screening (Bernardette Martinez-Rizo et al., 2024; Loncarevic et al., 2024). Thus, there is a pressing need for experimental systems that balance efficiency, mechanistic depth, and clinical relevance. In this regard, the zebrafish (*Danio rerio*) has rapidly emerged as a powerful model for natural products research (Li et al., 2017). Zebrafish offer several advantages: optical transparency, rapid development, and high fecundity, combined with strong conservation of immune cell types and inflammatory signaling pathways (approximately 70%–82% genomic homology with humans) (Howe et al., 2013; Kimmel et al., 1995; Sieber et al., 2019). Originally a cornerstone of developmental biology, zebrafish have evolved into a versatile platform for investigating immunology, inflammatory pathophysiology, and pharmacological screening (Zanandrea et al., 2020). In phytochemical research, they uniquely enable high-throughput screening of candidate compounds, simultaneous evaluation of efficacy and toxicity, and real-time imaging of immune cell dynamics and microenvironmental changes (Lin et al., 2022). Furthermore, zebrafish models provide tractable systems for studying host–pathogen interactions, offering novel perspectives on the regulation of inflammatory networks (Torraca and Mostowy, 2018).

Recent methodological advances have further expanded the utility of zebrafish. Integration with network pharmacology, CRISPR/Cas9-mediated gene editing, multi-omics analysis, and artificial intelligence (AI)-driven phenotypic profiling has provided deeper mechanistic insights and improved predictive accuracy. Humanized zebrafish xenograft models help bridge the gap between preclinical discovery and translational validation, advancing zebrafish as a platform for precision evaluation of plant-derived therapeutics (Silva Brito et al., 2022; Barros et al., 2008). Collectively, these innovations highlight the potential of zebrafish to serve as the basis of a mechanism-oriented natural product screening paradigm, accelerating the transition of phytochemicals from laboratory research to clinical application (Siekierska et al., 2019; Yan et al., 2019a).

**FIGURE 1:**
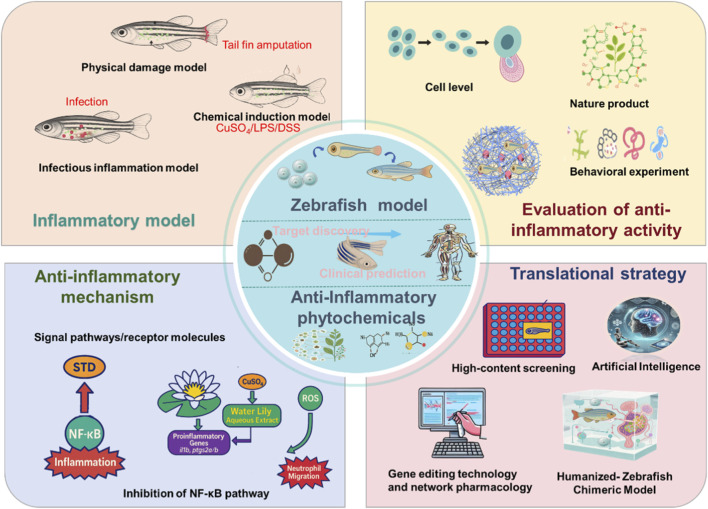
A multimodal zebrafish platform for anti-inflammatory phytochemical discovery. Upper left: Complementary inflammation models: physical injury, chemical induction, and infection. Upper right: Multi-level phenotypic screening through immune cell imaging and behavioral assessment of anti-inflammatory activity. Lower left: Mechanistic dissection using transgenic reporters, transcriptomics. Lower right: Translational pipeline integrating high-content screening, AI, network pharmacology, and humanized chimeras. Abbreviations: LPS, Lipopolysaccharide; DSS, Dextran Sulfate Sodium; NF-κB, Nuclear Factor Kappa-Light-Chain-Enhancer of Activated B Cells; ROS, Reactive Oxygen Species; STD, Stytontriterpene D.

Looking forward, the continued convergence of zebrafish biology with interdisciplinary technologies is poised to transform natural product pharmacology and anti-inflammatory drug discovery. By enabling efficient *in vivo* screening, robust mechanistic interrogation, and reliable translational prediction, zebrafish models offer a promising solution to one of the field’s most persistent challenges: bridging the gap between phytochemical discovery and clinical application (Smith, 2024). This review will therefore summarize recent advances in the application of zebrafish to the study of plant-derived anti-inflammatory agents, with particular emphasis on their roles in inflammation model construction, functional evaluation, activity screening, mechanistic elucidation, multimodal integration, and translational prediction. In doing so, we aim to chart a coherent path from “*in vivo* screening—mechanistic analysis—clinical translation” and to explore the future potential of zebrafish in precision medicine and personalized anti-inflammatory therapy (Figure 1).

## 2 Plant-derived anti-inflammatory chemicals: classification, disease association, and molecular mechanisms

### 2.1 Classification and characteristics of phytochemicals

**FIGURE 2:**
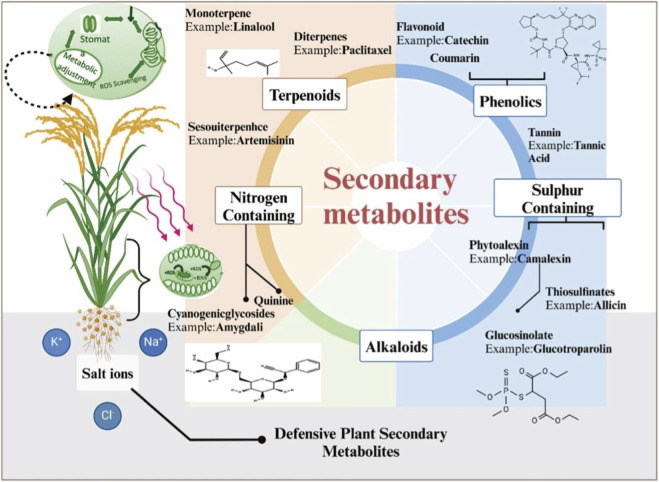
Structural diversity of key defensive plant specialized metabolites: alkaloids, phenolics, and terpenoids. Reprinted with the permission from Ref. (Ikram et al., 2025). Copyright @ 2025 Springer Nature.

Plants synthesize a vast array of specialized organic compounds, known as secondary metabolites, through biosynthetic pathways that are distinct from primary metabolism essential for growth and development (Figure 2) (Ikram et al., 2025). These phytochemicals, which have evolved under selective pressures, exhibit remarkable structural diversity and serve multifaceted biological roles. They play crucial defensive functions, protecting plants against pathogens, UV radiation, and abiotic stresses, while also constituting a rich source of lead compounds for pharmaceutical development (Leonov et al., 2015; Xu and Gaquerel, 2025). Based on their core structures and biosynthetic origins, major classes of phytochemicals with demonstrated anti-inflammatory properties include flavonoids, alkaloids, phenolics, and terpenoids.

Flavonoids, with a canonical C6–C3–C6 skeleton, comprise more than 6,500 identified molecules (Shen et al., 2022; Petrussa et al., 2013). Their abundant hydroxyl groups confer strong antioxidant and anti-inflammatory properties, supporting free radical scavenging, attenuation of oxidative stress, and regulation of inflammatory signaling through diverse pathways (Maroon et al., 2010; Choy et al., 2019; Treml and Šmejkal, 2016). Alkaloids, defined by nitrogen-containing heterocyclic structures, exhibit broad chemical diversity owing to varied substituents. In plants, they function as defense metabolites, while pharmacologically they display wide-ranging effects, including anti-inflammatory, antimicrobial, and immunomodulatory activities (Yele et al., 2025; Rahman, 2025). Phenolic compounds, unified by phenolic hydroxyl groups, play pivotal roles in plant defense. They directly scavenge reactive oxygen species (ROS) and inhibit pro-inflammatory signaling cascades, with some also demonstrating anticancer potential (Lee et al., 2015; Li X. et al., 2018; Bach et al., 2015). Terpenoids, assembled from isoprene (C5) units, possess exceptional structural variability and lipophilicity. These features underpin their diverse roles in plant growth regulation and defense, as well as their potent antimicrobial, anti-inflammatory, and immunomodulatory effects (Huang et al., 2022; Cho et al., 2017).

Collectively, these phytochemicals act through multi-target mechanisms, simultaneously influencing inflammatory signaling, redox homeostasis, and immune responses (Tasneem et al., 2019). This pleiotropic profile makes them especially well-suited for the prevention and treatment of complex chronic inflammatory disorders characterized by multifactorial etiologies and highly interconnected signaling networks (Table 1). Increasingly, phytochemicals are transitioning from traditional “adjuvant therapies” to front-line candidates next-generation anti-inflammatory drug development.

**TABLE 1:** Major classes of phytochemicals: Structural features, biological roles, and pharmacological activities.

Class	Core structural feature	Primary biological role in plants	Key pharmacological activities	Refs
Flavonoid	C6-C3-C6 backbone; phenolic -OH groups	Ubiquitous secondary metabolites	Anti-inflammatory; antioxidant	Petrussa et al. (2013), Shen et al. (2022), Choy et al. (2019), Maroon et al. (2010)
Alkaloid	N-heterocyclic core; variable substituents	Defense against environmental stress	Anti-inflammatory; antimicrobial; broad-spectrum bioactivity	Rahman (2025), Yele et al. (2025)
Phenolic	Phenolic-OH groups	Key components in plant defense systems	Anti-inflammatory; anticancer	Bach et al. (2015), Lee et al. (2015), Li et al. (2018b)

### 2.2 Molecular mechanisms of anti-inflammatory action of phytochemicals

The anti-inflammatory activity of phytochemicals is largely mediated through precise regulation of three interconnected signaling axes: NF-κB, MAPK, and Nrf2 (Sadiq et al., 2024; Shin et al., 2020). Together, these cascades orchestrate the initiation and resolution of inflammation.

**FIGURE 3:**
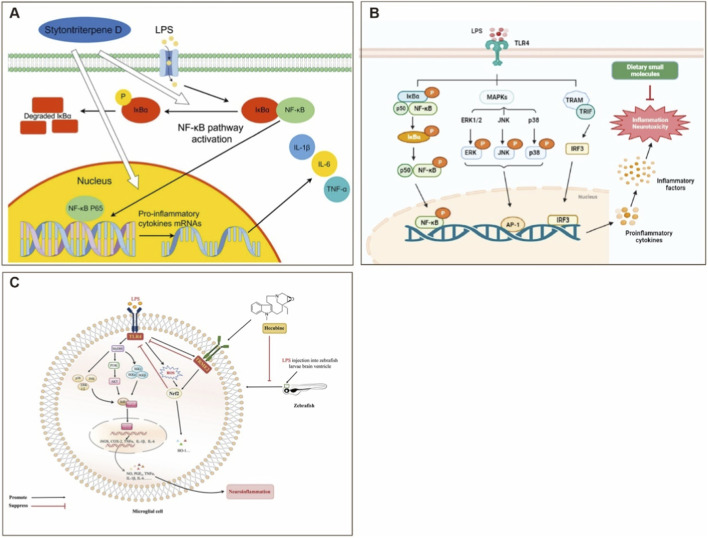
Molecular mechanisms of natural products and dietary small molecules in modulating inflammatory signaling pathways in zebrafish models **(A)** Stytontriterpene D inhibits LPS-induced NF-κB activation by preventing IκBα phosphorylation and degradation, thereby blocking P65 nuclear translocation and transcription of pro-inflammatory cytokines (IL-1β, IL-6, TNF-α) Reprinted with the permission from Ref. (Yu et al. 2025). Copyright © Frontiers **(B)** Dietary Small molecules exert pleiotropic anti-inflammation effects by convergently targeting TLR4 downstream cascades. LPS engagement activates MyD88-dependent (IKK-NF-κB, MAPK-AP-1) and TRIF-dependent (IRF3) axes, leading to nuclear translocation of transcription factors and pro-inflammatory gene expression. Dietary compounds antagonize these pathways at multiple nodal points to attenuate inflammation-induced neurotoxicity. Reprinted with the permission from Ref. (Balakrishnan et al., 2024). Copyright © 2024 Elsevier **(C)** Hecubine activates TREM2 to orchestrate dual anti-inflammatory and antioxidant defense. TREM2 activation suppresses TLR4-MyD88-IKK-NF-κB/MAPK signaling (red inhibitory arrows) while promoting PI3K-Akt-Nrf2 nuclear translocation (green arrows) and antioxidant gene (HO-1) expression. TREM2 knockdown abolishes these effects, confirming requisite target engagement. *In vivo* efficacy was demonstrated in a zebrafish larval brain ventricle microinjection model of LPS-induced neuroinflammation. Reprinted with the permission from Ref. (Li et al., 2024). Copyright © 2024 Elsevier. Arrow legend: Solid arrows denote positive signal flow/translocation; blunt-ended arrows indicate inhibitory actions; “P” symbolizes phosphorylation; red arrows/bars represent Hecubine-mediated pathway suppression; green arrows denote antioxidant pathway activation. Abbreviations: Abbreviations: LPS, lipopolysaccharide; TLR4, toll-like receptor 4; IKK, IκB kinase; MAPK, mitogen-activated protein kinase; ERK1/2, extracellular signal-regulated kinase 1/2; JNK, c-Jun N-terminal kinase; AP-1, activator protein-1; IRF3, interferon regulatory factor 3; TREM2, triggering receptor expressed on myeloid cells 2; Nrf2, nuclear factor erythroid 2-related factor 2; HO-1, heme oxygenase-1; CETSA, cellular thermal shift assay.

NF-κB is a central regulator of pro-inflammatory gene expression. In a testosterone propionate-induced rat model of prostatic hyperplasia, curcuma oil suppressed 5α-reductase expression and p65 phosphorylation, thereby blocking NF-κB activation, reducing inflammation, and delaying disease progression (Wang S. et al., 2021). Stytontriterpene D Suppresses Lipopolysaccharide (LPS)-Induced Inflammatory Response by Inhibiting the NF-κB Signaling Pathway (Figure 3A) (Yu et al., 2025). Similarly, Quzhou *Fructus Aurantii* Extract (QFAE) attenuated NF-κB signaling in LPS-stimulated macrophages and a mouse acute lung injury model, significantly lowering Tumor Necrosis Factor-Alpha (TNF-α), IL-6, and IL-1β levels, while enhancing IL-10 expression and reducing tissue pathology (Li et al., 2020).

The MAPK pathway, which crosstalks extensively with NF-κB, is also a critical target (Sung et al., 2024). *Lycium ruthenicum* ethanol extract (LRE) mitigated colonic damage, restored immune homeostasis, and inhibited NLRP3 inflammasome assembly by suppressing MAPK activity in DSS (dextran sulfate sodium)-induced colitis mice (Zong et al., 2020). Olive oil polyphenols blocked aberrant IL-6/IL-8 expression induced by oxidized sterols through MAPK–NF-κB signaling, thereby preserving intestinal homeostasis (Serra et al., 2018). Small molecules mainly inhibit the secretion and expression of related inflammatory factors through MAPK pathways (Figure 3B) (Balakrishnan et al., 2024).

Nrf2, a master regulator of antioxidant defense, represents the third major axis (Wang and Yang, 2025). *Myristica fragrans* (nutmeg) extract, acting through the Akt/JNK–Keap1–Nrf2–HO-1 cascade, simultaneously reduced pro-inflammatory cytokines (TNF-α, IL-1β, IL-6) and oxidative stress markers (MDA, MPO), while enhancing IL-10, SOD, and GSH-Px activities (Li et al., 2025). *Codonopsis pilosula* (Dangshen) extract demonstrated dual regulation in a chronic obstructive pulmonary disease model: activating Nrf2 while suppressing NF-κB, as indicated by reduced p-p65/p65 and p-IκB-α/IκB-α ratios, leading to improvements in both inflammation and oxidative stress (Chen et al., 2024). Via direct targeting of TREM2 (triggering receptor expressed on myeloid cells 2), Hecubine alleviated neuroinflammation and oxidative stress in both *in vivo* (4 dpf zebrafish larvae) and *in vitro* (BV-2 microglial cells) models, by modulating the Nrf2 and TLR4 signaling pathways (Figure 3C) (Li et al., 2024).

Overall, the anti-inflammatory mechanisms of phytochemicals are inherently multidimensional, operating through NF-κB inhibition, MAPK modulation, and Nrf2 activation (Saleh et al., 2021; Zhang et al., 2019; Gong et al., 2020). This multi-target, multi-pathway paradigm overcomes the limitations of single-target drugs and highlights their potential for precision intervention in inflammatory disease.

### 2.3 Role of phytochemicals in inflammation-related diseases

Plant-derived phytochemicals are undergoing a paradigm shift from being perceived as mere “adjunct therapies” to emerging as front-runner candidates for next-generation anti-inflammatory drug development (Wongrakpanich et al., 2018). For instance, Auraptene, a bioactive compound derived from citrus fruits, has progressed beyond traditional use to enter clinical trials aimed at preventing Alzheimer’s disease, showcasing its translational potential in neuroinflammation and neurodegeneration (Bayo et al., 2025).

Phytochemicals display broad therapeutic potential in inflammation-associated diseases, including cardiovascular, metabolic, and neoplastic disorders (Fang et al., 2023). Quercetin scavenges reactive oxygen species such as superoxide anions and hydroxyl radicals, while enhancing antioxidant enzyme expression via activation of the Keap1/Nrf2 pathway, thereby protecting vascular endothelium from oxidative injury and preserving vascular function (Li et al., 2021). Additionally, bioactive constituents from garlic, onion, and turmeric modulate lipid metabolism through PPARγ activation, further contributing to cardiovascular protection (Lin et al., 2017; Doi et al., 2022; Lin et al., 2015).

In metabolic diseases, phytochemicals similarly exert multi-target regulatory effects (He et al., 2024; Maruca et al., 2017). Tea polysaccharides improved lipid metabolism and remodeled gut microbiota composition in diabetic models; mechanistic studies linked these effects to modulation of bile acid metabolism and insulin signaling (Wu et al., 2022). *Dioscorea opposite* polysaccharides lowered blood glucose, enhanced insulin secretion, and improved islet function in diabetic rats, through mechanisms involving lipid peroxide reduction and ROS scavenging, thereby preserving cellular integrity (Fan et al., 2015).

In cancer prevention and therapy, the pleiotropy of phytochemicals is particularly striking (George et al., 2021; Rizeq et al., 2020). *Asparagus* extract inhibited proliferation and glycolysis in endometrial cancer cells, induced stress-mediated apoptosis, caused G1-phase arrest, and sensitized cells to cisplatin, underscoring its adjuvant potential (Fang et al., 2024). Curcumin and epigallocatechin gallate (EGCG) inhibit tumor growth and inflammation by targeting COX-2 and NF-κB signaling (Rizeq et al., 2020; Limanaqi et al., 2020). Collectively, these findings highlight the capacity of phytochemicals to modulate disease progression through multi-layered and multi-pathway mechanisms, offering new therapeutic opportunities beyond the limitations of conventional single-target drugs.

## 3 Zebrafish inflammation models and construction strategies

The research and development of plant-derived anti-inflammatory agents face persistent challenges in achieving efficient compound screening, robust activity validation, and mechanistic elucidation. The zebrafish, which uniquely integrates the systemic complexity of *in vivo* models with the scalability of experimental platforms, has emerged as a pivotal bridge between *in vitro* assays and mammalian systems. Owing to its versatility and translational relevance, zebrafish is increasingly recognized as an indispensable tool in anti-inflammatory research.

### 3.1 Advantages of zebrafish inflammation models

The zebrafish, with its transparent embryos, rapid development, and well-characterized genetic background, has become a widely adopted model for dissecting inflammatory mechanisms and evaluating drug effects in recent years (Larijani et al., 2022). Unlike conventional *in vitro* systems, which lack physiological complexity, or mammalian models, which are costly and low-throughput, zebrafish uniquely integrate experimental accessibility with *in vivo* relevance. Their optical transparency enables real-time, non-invasive imaging of immune behaviors such as neutrophil migration, while their fecundity and small size support large-scale phenotypic screens in multi-well formats—a capacity not feasible in rodents. Although medaka and *Xenopus* offer certain advantages, they are constrained by fewer genetic tools or slower development, rendering zebrafish superior in genetic tractability and immunological assay versatility (Chowdhury et al., 2022; Wang and Cao, 2021). Thus, zebrafish provide an optimal balance of physiological conservation, experimental efficiency, and imaging capability for inflammation research.

Mouse models provide high anatomical and pathological conservation with humans, making them indispensable for modeling complex chronic diseases and enabling behavioral or immunological studies (Gonzalez-Ramos et al., 2023; Brown et al., 2017; Ack et al., 2016; Koechling et al., 2010). However, they present notable limitations, including high maintenance costs, long generation times (∼3 months to sexual maturity), reduced feasibility for large-scale screening, constraints in high-resolution live imaging (often requiring sacrifice or invasive methods), and increasingly stringent ethical considerations (Brown et al., 2017). By contrast, zebrafish overcome many of these limitations. Their high fecundity (200–300 eggs per female per week), rapid embryonic development (major organs form within 24 h), and compatibility with microtiter plates (e.g., 96-well formats) significantly accelerate high-throughput screening, while reducing compound consumption and experimental cost. Although *in vitro* systems offer controllability, short experimental cycles, and are amenable to high-throughput screening (d'Amora and Giordani, 2018; Kobar et al., 2021), they lack the tissue microenvironment, cell–cell interactions, systemic feedback, and metabolic capacity necessary for accurately predicting *in vivo* efficacy and toxicity (Koechling et al., 2010; d'Amora and Giordani, 2018; Kobar et al., 2021; Verma SK. et al., 2024).

**FIGURE 4:**
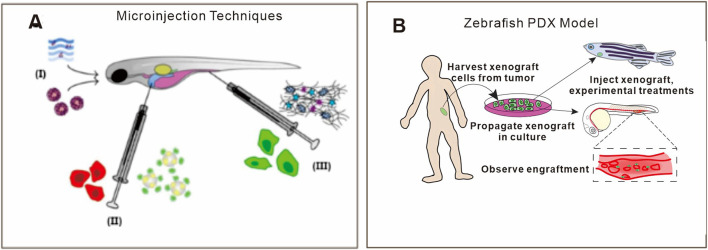
Representative applications of zebrafish models in biomedical research **(A)** Microinjection techniques facilitate the introduction of nucleic acids, small molecules, and diverse cell types into embryos, allowing dissection of gene regulatory networks and disease mechanisms. Reprinted with the permission from Ref. (Cheng et al., 2016). Copyright © 2016 Wiley-VCH **(B)** Patient-derived xenograft (PDX) zebrafish models, established by transplanting patient tumor cells into embryos, enable real-time monitoring of tumor engraftment, progression, and therapeutic responses, offering a platform for cancer research and personalized drug evaluation. Reprinted with the permission from Ref. (Molina et al., 2021). Copyright © 2021 Wiley-VCH.

The optical transparency of zebrafish embryos and larvae enables real-time tracking of neutrophil and macrophage migration, aggregation, and phagocytosis at single-cell resolution—difficult to achieve in mammalian models (Gonzalez-Ramos et al., 2023; Brown et al., 2017; Ack et al., 2016). Importantly, comparative genomics reveal 70%–82% homology with humans in key inflammatory pathways such as NF-κB and NLRP3, establishing a reliable molecular basis for elucidating phytochemical mechanisms (Barbazuk et al., 2000; He et al., 2024; Sommer et al., 2020). These imaging advantages allow cost-efficient, dynamic monitoring of immune cell behavior, inflammatory progression, and tissue repair processes (Habjan et al., 2024; Zon and Peterson, 2005; Mukherjee et al., 2024). Moreover, in accordance with EU Directive 2010/63/EU, zebrafish embryos prior to 120 h post-fertilization (hpf) are exempt from animal ethics review, facilitating ethically compliant large-scale drug screening. Evaluating diverse administration paradigms in zebrafish larvae is essential for elucidating the uptake, transport, metabolism, and efficacy of therapeutic agents, including both small molecule drugs and cell-based therapies (Figure 4A) (Cheng et al., 2016). Patient-derived xenograft (PDX) zebrafish models, established by transplanting patient tumor cells into embryos, enable real-time monitoring of tumor engraftment, progression, and therapeutic responses, offering a platform for cancer research and personalized drug evaluation (Figure 4B) (Molina et al., 2021). Drug administration in zebrafish is highly versatile, encompassing immersion, yolk sac or circulatory injection, and oral gavage (Fazio et al., 2020; Dang et al., 2016; Chaoul et al., 2023). Immunodeficient strains (e.g., *prkdc* −/−; *il2rga* −/−) allow transplantation of human immune cells or organoids, creating “humanized” platforms to investigate phytochemical immunomodulation (Yan et al., 2019b). While transgenic lines such as *Tg(mpx:GFP)* have enabled dynamic, real-time visualization of neutrophil migration and infiltration during inflammatory responses (Renshaw et al., 2006; Ellett et al., 2011; Hall et al., 2007), several limitations warrant consideration. First, developmentally regulated expression of the fluorescent reporter can give rise to background fluorescence that may confound the interpretation of inflammation-specific signals. Second, fluorescence intensity serves only as an indirect proxy for cellular recruitment and may not quantitatively correspond to the transcriptional activity of relevant genes. Third, in adult organisms, diminished optical transparency restricts high-resolution imaging of deep tissues, thereby limiting the applicability of these models for *in vivo* studies at later developmental stages.

Zebrafish share ∼80% of human disease genes, and their early embryos lack adaptive immunity, permitting xenotransplantation of human tumor cells without immunosuppression (Brown et al., 2017; d'Amora and Giordani, 2018; Molina et al., 2021). In summary, zebrafish uniquely combine high-throughput capability, superior live imaging, cost-effectiveness, and strong immunological conservation with humans, thereby providing a dynamic and translationally relevant platform for anti-inflammatory drug discovery.

Beyond these native advantages, the zebrafish platform is undergoing a paradigm shift—from a passive disease model to an active, technology-integrated discovery engine. Recent convergence with microfluidics, artificial intelligence, gene editing, network pharmacology, and humanized xenograft systems has fundamentally expanded its translational capacity. These integrations transform zebrafish from a high-throughput screening tool into a multimodal platform capable of mechanistic interrogation, predictive pharmacokinetics, and patient-relevant efficacy modeling. Unlike conventional cellular or mammalian systems, zebrafish now uniquely enable real-time multi-omic profiling, spatiotemporal immune tracking, and cross-species validation within a single, scalable framework. This technological evolution underpins the multimodal architecture detailed in Section 4 and Figure 6, positioning zebrafish as a central bridge between phytochemical discovery and clinical application.

### 3.2 Zebrafish inflammation models construct strategies

Zebrafish inflammation models are commonly established through chemical, physical, and infection-based approaches, each presenting unique advantages for investigating the mechanisms of action of phytochemicals (Zanandrea et al., 2020). Acute inflammation—typically induced by tail fin amputation or chemical exposure—is characterized by a robust immune response initiated within hours of injury and subsequent resolution within 24–48 h, in contrast to chronic inflammation involving persistent immune stimulation and tissue damage (Xie et al., 2020; Audira et al., 2022). The models covered in this review are exclusively acute inflammatory models. These models are rapidly inducible, technically facile, and exhibit robust, reproducible phenotypes (Table 2).

**TABLE 2:** Application of zebrafish inflammation models in studying the anti-inflammatory mechanisms of plant compounds.

Model Category	Specific Model	Key Parameters	Phenotypes/Outcomes	Monitoring Methods	Primary Applications	Key Advantages	Refs
Chemical Induction	LPS (Systemic)	• Concentration: 10 μg/mL • Duration: 72 h • Pathway: TLR4/NF-κB	↑ TNF-α, IL-1β, IL-6 ↑ ROS	qRT-PCR (cytokines); ROS assay	Anti-inflammatory compounds (e.g., fucoidan, flavonoids)	High reproducibility; Neuro/Gut models	Balkrishna et al. (2021), Szumlak et al. (2024)
	(CuSO_4_ (oxidative stress)	• Concentration: 10 μM • Duration: 15 min–2 h	Neutrophil migration to injury site (peak: 2 h)	Live imaging (neutrophil trafficking)	HTS of antioxidant/anti-inflammatory agents	Non-invasive; rapid onset HTS-compatible	Mao et al. (2025), Renshwa et al. (2010), Xie et al. (2020)
	DSS (gut dysfunction)	• Concentration: 0.5% • Duration: 72 h	Intestinal epithelial damage; ↑ IL-1β, IL-8, MMP9; ↓ SOD	Histopathology; qRT-PCR SOD assay	Phytochemicals for gut barrier protection	Recapitulates IBD-like pathology	Li et al. (2022)
Physical injury	Tail fin amputation	• Stage: Embryo to adult • Site: Tail fin • Timecourse: Neutrophils (peak: 4 hpi); macrophages (peak: 6 hpi)	Wound-induced inflammation	*Tg(mpx:GFP)* (neutrophils); *Tg(mpeg1:mCherry)* (macrophages)	HTS of natural compounds; inflammation-resolution dynamics	Spatiotemporal resolution Real-time immune tracking	Li et al. (2012), Xie et al. (2020), Lama et al. (2022)
Infection models	*A. hydrophila* (sepsis-like)	• Route: Microinjection/Immersion • Phenotypes: Hemorrhage, hepatosplenomegaly	Systemic infection	Confocal imaging (bacterial dissemination); qPCR (immune genes)	Dual-activity (antibacterial + anti-inflammatory) compounds	Mimics natural infection Human sepsis relevance	Saraceni et al. (2016)
	*P. aeruginosa* (drug-resistant)	• Strain: PAO1 • Method: Immersion post-amputation • Timecourse: Macrophage recruitment (peak: 24 hpi)	Acute drug-resistant infection	Phagocytosis assays; bacterial load quantification	Anti-infectives against multidrug-resistant pathogens	Rapid acute infection (20 hpi); host-pathogen interaction studies	Nogaret et al. (2021), Yang et al. (2025)

↑, positive regulation; ↓, negative regulation.

Abbreviations: LPS, Lipopolysaccharide; ROS, Reactive Oxygen Species; HTS, High-Throughput Screening; DSS, Dextran Sulfate Sodium; SOD, Superoxide Dismutase.

Chemically induced models are the most widely employed for high-throughput screening. Intravenous or intraperitoneal injection of lipopolysaccharide (LPS) activates the zebrafish TLR4/NF-κB pathway, eliciting systemic inflammatory responses that recapitulate pathological conditions such as neuroinflammation and enteritis. Exposure of zebrafish larvae to 10 μg/mL LPS for 72 h significantly upregulates pro-inflammatory cytokines, including TNF-α, IL-1β, and IL-6, and induces marked reactive oxygen species (ROS) production (Szumlak et al., 2024; Balkrishna et al., 2021). This model has been extensively utilized to evaluate natural compounds such as fucoidan, flavonols, and polyphenols (Wang et al., 2022; Molagoda et al., 2021; Wang et al., 2025).

Copper sulfate (CuSO_4_) immersion induces oxidative stress and neutrophil-driven inflammation, wherein neutrophil migration is observable within 15 min and reaches maximal accumulation by 2 h. This acute, non-invasive model is highly amenable to high-throughput compound screening (Xie et al., 2020; Renshaw and Ingham, 2010; Mao et al., 2025). Similarly, dextran sulfate sodium (DSS) induces intestinal epithelial injury; treatment with 0.5% DSS for 72 h produces marked epithelial disruption, elevates the expression of IL-1β, IL-8, and MMP9, and decreases superoxide dismutase (SOD) activity, thereby establishing a model of zebrafish intestinal dysfunction (Li et al., 2022).

Infection-induced models offer unique insights into host–pathogen interactions. *Aeromonas hydrophila* infection in 3-day-post-fertilization (dpf) embryos, induced via microinjection or immersion, mimics natural infection routes and facilitates the assessment of immune responses through confocal imaging and qRT-PCR (Saraceni et al., 2016). Similarly, immersion of amputated larvae in *Pseudomonas aeruginosa* strain PAO1 induces acute infection within 20 h, characterized by rapid neutrophil and macrophage recruitment (Yang et al., 2025; Nogaret et al., 2021). These models are particularly useful for evaluating phytochemicals with dual antibacterial and anti-inflammatory activity.

Physical injury models, particularly tail fin amputation, are classical tools for dissecting acute inflammation and tissue repair with high spatiotemporal resolution. Neutrophil migration peaks at ∼4 h post-amputation, followed by macrophage recruitment and dominance in wound repair from ∼6 h onward (Xie et al., 2020; Li et al., 2012). Transgenic lines such as *Tg(mpx:GFP)* and *Tg(mpeg1:mCherry)* allow precise real-time monitoring of immune cell dynamics (Lama et al., 2022). This model is well established for screening both natural products and FDA-approved drug libraries (Xie et al., 2020; Xiong et al., 2022).

Despite the widespread adoption of zebrafish inflammation models, inter-laboratory variability remains poorly characterized. For LPS-induced models, factors such as serotype, vehicle, injection site, and developmental stage can precipitate substantial variations (Balkrishna et al., 2021). CuSO_4_ protocols are similarly heterogeneous regarding concentration, exposure duration, and recovery time, factors which directly modulate neutrophil recruitment kinetics (Xie et al., 2020). This lack of uniformity compromises meta-analyses and hinders cross-study comparisons of phytochemical efficacy. Consequently, the establishment of rigorous standards is essential to standardize operational procedures and guarantee result reproducibility.

In summary, zebrafish inflammation models constitute a robust experimental repertoire for anti-inflammatory research. Chemically induced systems enable efficient high-throughput screening; physical injury models provide unparalleled resolution of immune cell dynamics; and infection-based models facilitate the evaluation of compounds with combined antimicrobial and anti-inflammatory properties. Future directions include the integration of zebrafish models with omics technologies, humanized transplantation systems, and AI-assisted image analysis, which is poised to further expand their utility in the mechanistic dissection and clinical translation of plant-derived anti-inflammatory therapeutics.

### 3.3 Functional and mechanistic studies of phytochemical anti-inflammatory based on zebrafish models

#### 3.3.1 Anti-inflammatory functions of phytochemical anti-inflammatory

Zebrafish inflammation models have evolved into a multidimensional evaluation system encompassing whole-organism behavior, tissue-level pathology, and molecular mechanisms, thus providing a robust *in vivo* platform for systematic assessment of the anti-inflammatory efficacy of phytochemicals (Xie et al., 2020).

At the whole-organism level, behavioral monitoring and histological analysis constitute the foundation of pharmacodynamic evaluation. In neuroinflammation studies, automated tracking systems such as Zebrabox enable simultaneous monitoring of up to 96 larvae in multi-well plates or groups of zebrafish, generating quantitative data on trajectory maps, swimming speed, movement distance, and path complexity (Castro-Sepulveda et al., 2025; Chen et al., 2025b; Ravikumar et al., 2025). For example, ethanol leaf extract of *Orthosiphon stamineus* markedly improved pentylenetetrazole (PTZ)-induced seizures in zebrafish, with high-dose effects surpassing those of diazepam. Mechanistically, these effects were associated with downregulation of NF-κB, NPY, and TNF-α (Choo et al., 2018). Likewise, essential oil (EO) from *Cymbopogon citratus* alleviated PTZ-induced seizures in adult zebrafish, reduced brain levels of malondialdehyde (MDA) and nitric oxide (NO), and enhanced glutathione (GSH) and catalase (CAT) activity, thereby demonstrating neuroprotective potential (Hacke et al., 2021). At the molecular level, zebrafish also provides quantifiable evaluation methods. For example, *Tribulus terrestris* extract (BJL) significantly inhibited neutrophil migration and aggregation induced by tail fin transection or LPS. qRT-PCR results showed that BJL downregulated the expression of iNOS and NF-κB, thereby reducing the production of NO and inflammatory cytokines (Zhao et al., 2021). Collectively, behavioral and histological endpoints provide intuitive and reliable indicators for evaluating inflammatory responses and drug interventions in zebrafish.

At the cellular level, the intrinsic optical transparency of zebrafish embryos enables direct visualization of immune cell dynamics. In particular, transgenic lines such as *Tg(mpx:GFP)* allow real-time monitoring of neutrophil migration (Wang J. et al., 2024). Using this model, the methanol extract of the Antarctic lichen *Amandinea* sp. Was shown to attenuate LPS-induced inflammation by inhibiting the release of pro-inflammatory cytokines (IL-6, TNF-α) and mediators (iNOS, COX-2), decreasing ROS accumulation, and downregulating the expression of inflammatory genes in a tail transection-induced inflammation assay, thereby confirming its potent anti-inflammatory activity (Kim et al., 2020). The combination of optical transparency with transgenic technology thus provides unique advantages for dissecting immune cell behavior and drug effects on inflammatory cellular responses *in vivo*.

Molecular-level analyses remain indispensable for elucidating inflammatory signaling pathways and drug targets. For instance, extract of BJL significantly inhibited neutrophil migration and aggregation induced by caudal fin transection or LPS challenge. qRT-PCR analysis demonstrated that BJL downregulated iNOS and NF-κB expression, leading to reduced NO production and suppression of pro-inflammatory cytokine expression (Zhao et al., 2021). These findings provide mechanistic insights into its anti-inflammatory actions. In conclusion, molecular-level analyses represent a core approach for delineating inflammatory pathways and intervention mechanisms in zebrafish.

#### 3.3.2 Anti-inflammatory mechanisms of polyphenols and flavonoids

The zebrafish model provides a powerful and efficient platform for elucidating the anti-inflammatory mechanisms of polyphenols and flavonoids (Yang et al., 2014). For example, *xanthohumol* markedly attenuates inflammatory responses induced by LPS and CuSO_4_ in zebrafish. Pretreatment with 2 μg/mL *xanthohumol* prior to LPS exposure significantly improved larval survival (>80% compared with 25% in the LPS-only group), exceeding the efficacy of the positive control ibuprofen (Szumlak et al., 2024). Mechanistic studies demonstrated that *xanthohumol* suppresses *IL-1*β expression, enhances *IL-10* production, and mitigates CuSO_4_-induced oxidative stress. Similarly, ethanol extracts of chrysanthemum stems and leaves, tested in a DSS-induced inflammatory bowel disease model, downregulated *IL-1*β, *IL-8*, and *MMP9* while enhancing superoxide dismutase (SOD) activity, thereby alleviating intestinal inflammation (Li et al., 2022).

Quercetin (50 μM) ameliorated developmental retardation in zebrafish larvae exposed to *Botrytis cinerea* spores by reducing abnormal apoptosis, strengthening antioxidant defenses, suppressing inflammatory responses, repairing intestinal architecture, and restoring peristaltic activity. Mechanistic analysis revealed that quercetin activates the *Keap1–Nrf2* pathway, upregulates downstream antioxidant genes (*nrf2*, *ho-1*, *sod*, *cat*), and increases SOD and catalase (CAT) activity, thereby eliminating excessive reactive oxygen species (ROS) and malondialdehyde (MDA). Furthermore, *quercetin* inhibits apoptosis by downregulating pro-apoptotic factors (*bax*, *p53*, *caspase-3*, *caspase-9*) while upregulating the anti-apoptotic gene *bcl-2* (Shi et al., 2023).

Collectively, these findings demonstrate that polyphenols and flavonoids act through a synergistic multi-target mode of action— “anti-inflammatory + antioxidant + anti-apoptotic”—which is more suitable for addressing the complex pathological processes of chronic inflammation compared to conventional single-target drugs.

#### 3.3.3 Anti-inflammatory mechanisms of alkaloids and glycosides

Zebrafish models also provide unique advantages in dissecting the anti-inflammatory activities of alkaloids and glycosides, including their potential epigenetic regulatory effects. *Stytontriterpene D* (STD), for instance, significantly reduced inflammatory cell recruitment in tail fin amputation and CuSO_4_-induced models by inhibiting the expression of *NF-κB p65* and *IκBα* (Yu et al., 2025). In an LPS-induced neuroinflammation model established by microinjecting LPS into the brains of four dpf larvae, pretreatment with *hecubine* for 24 h markedly reversed the LPS-induced upregulation of pro-inflammatory mediators, including inducible nitric oxide synthase (*iNOS*), tumor necrosis factor-α (*TNF-*α), interleukin-6 (*IL-6*), and *IL-1*β (Li et al., 2024).

More recently, *tuberoside H* (TH), isolated from *Marsdenia tenacissima*, exhibited significant anti-inflammatory effects in the transgenic zebrafish line *Tg(lyz:EGFP)*. At concentrations of 0.05–0.15 mg/mL, TH inhibited CuSO_4_-induced immune cell migration, suppressed *NF-κB* and *p38 MAPK* signaling, reduced the expression of *TNF-*α, *IL-1*β, and *IL-8*, while enhancing secretion of the anti-inflammatory cytokine *IL-10* (Li JJ. et al., 2018). Likewise, *herpotriquinones A* inhibited inflammatory cell infiltration in a concentration-dependent manner in a CuSO_4_-induced neuroinflammation model. Western blot analysis confirmed blockade of phosphorylation within the *NF-κB* and *MAPK* (ERK/JNK) pathways, leading to reduced pro-inflammatory cytokine release and providing novel insights into natural products targeting neuro-inflammation (Zhai et al., 2024).

In addition, Evodiamine alleviates intestinal inflammation by modulating the NF-κB/STAT3 and IRF5 pathways to mitigate intestinal tissue injury in DSS-induced (Fan et al., 2025). Together, these results highlight the distinctive value of zebrafish models for elucidating how alkaloids and glycosides modulate inflammation through multiple signaling pathways, including *NF-κB*, *TREM2*, *Nrf2*, and *JAK/STAT*. This multi-target paradigm provides a systematic framework for the discovery and development of novel anti-inflammatory drugs.

#### 3.3.4 Anti-inflammatory mechanism studies of other active constituents

The wide array of zebrafish inflammatory models—including CuSO_4_ exposure, tail fin amputation, DSS-induced inflammatory bowel disease (IBD), and LPS-induced neuroinflammation—offers an ideal platform for systematically investigating the pleiotropic anti-inflammatory effects of plant-derived extracts. For example, *Ludwigia repens* extract enhanced SOD and CAT activity, mitigating oxidative stress induced by CuSO_4_ exposure and tail fin amputation (Rajiv et al., 2024). Likewise, ethanol extracts from *Clerodendrum cyrtophyllum* leaves protected against CuSO_4_-induced oxidative stress by downregulating HSP70 and GADD45BB while upregulating antioxidant responses (Nguyen et al., 2020). *Angelicain B* also exhibited strong anti-inflammatory activity, significantly inhibiting ROS overproduction and downregulating *TNF-*α and *IL-6* mRNA levels in a CuSO_4_-induced zebrafish model (Zhang et al., 2025).

Collectively, these findings confirm that zebrafish are highly effective for elucidating the multi-target mechanisms of diverse phytochemicals, encompassing regulation of redox balance, modulation of inflammatory mediators, and control of central signaling pathways such as *NF-κB*, *MAPK*, and *Nrf2* (Table 3). Importantly, this research transcends the validation of single active molecules; it highlights the systems-level actions of plant extracts characterized by “multi-component, multi-target, multi-pathway” interactions.

**TABLE 3:** Mechanistic insights into anti-inflammatory effects of phytochemicals using zebrafish translational models.

Compound class and source	Key anti-inflammatory Mechanism(s)	Critical evidence in zebrafish models	Model-specific advantages and techniques	Ref.
Xanthohumol	↓ IL-1β, ↑ IL-10; ↓ oxidative stress	↑ survival (80% vs. 25% LPS control); ↓ Cell damage in CuSO_4_ model	LPS/CuSO_4_-induced systemic inflammation; survival analysis	Szumlak et al. (2024)
*Chrysanthemum* stem-leaf extract	↓ IL-1β, IL-8, MMP9; ↑ SOD activity	↓ inflammation in DSS-induced IBD model; improved intestinal pathology	DSS-induced inflammatory bowel disease (IBD) Cytokine profiling	Nguyen et al. (2020)
Quercetin	↓ *bax*, *p53*, caspase3/9; ↑ bcl-2; activation of Keap1-Nrf2 pathway → ↑ *nrf2*, ho-1, *sod*, *cat*; ↑ SOD/CAT activity	↓ apoptosis and ROS/MDA; restored gut morphology and peristalsis in *Botrytis cinerea*-induced model	Infection-induced developmental disorder Live imaging of gut function	Shi et al. (2023)
Alkaloids and Glycosides				
Stytontriterpene D (STD)	Inhibition of NF-κB pathway: ↓ *nfκb p65*, *iκbα* mRNA	↓ inflammatory cell recruitment in tail-amputation and CuSO_4_ models	Tail amputation + CuSO_4_-induced inflammation Cell migration assays	Yu et al. (2025)
Hecubine	Activation of TREM2 and Nrf2; ↓ iNOS, TNF-α, IL-6, IL-1β	Reversed LPS-induced neuroinflammation in larval head	LPS-induced neuroinflammation Targeted brain injection	Li et al. (2024)
Other active constituents				
*Jussiaea repens* methanol extract	↑ CAT/SOD activity	↓ inflammation and oxidative stress in CuSO_4_/tail-amputation models	Multi-model validation (chemical + physical injury)	Rajiv et al. (2024)
*Clerodendrum cyrtophyllum* ethanol extract	↓ *hsp70*, *gadd45 b b*; ↑ SOD; ↓ cox-2, *pla2*, *c3a*, *mpo*, il-1β, il-8, *tnf-α*	↓ ROS (fluorescence probe); protected against CuSO_4_-induced oxidative stress and inflammation in larvae	Real-time ROS detection (live imaging) Multigene inflammatory panel	Nguyen et al. (2020)
Angelicain B	↓ ROS, ↓ *tnf-α*, il-6 mRNA	Inhibited CuSO_4_-induced ROS overproduction and pro-inflammatory gene expression	ROS-sensitive fluorescent probes; qPCR validation	Zhang et al. (2025)

↑, positive regulation; ↓, negative regulation.

Abbreviations: IL, Interleukin; LPS, Lipopolysaccharide; SOD, Superoxide Dismutase; ROS, Reactive Oxygen Species; NOS, Inducible Nitric Oxide Synthase; qRT-PCR, Quantitative Real-Time Polymerase Chain Reaction; IBD, Inflammatory Bowel Disease.

In conclusion, zebrafish models have emerged as a pivotal platform in plant-derived anti-inflammatory research. They enable early-stage high-throughput screening, mechanistic dissection via transgenic imaging, and multi-omics integration. Compared with cell-based systems, zebrafish provide a closer approximation to *in vivo* physiology, while offering greater efficiency, lower cost, and enhanced suitability relative to mammalian models for exploring complex mechanisms. Looking ahead, advances in AI-driven image analysis, spatial transcriptomics, and multimodal omics will further expand the role of zebrafish as a Frontier tool for personalized drug discovery and precision medicine, progressively bridging the gap between theoretical exploration and clinical translation of plant-derived compounds.

### 3.4 Does zebrafish offer unique advantages for plant-derived anti-inflammatory agents?

A central question in this review is whether the attributes of the zebrafish model are particularly suited to the study of plant-derived compounds or are broadly applicable to anti-inflammatory drug discovery. We propose that zebrafish offer three distinct advantages with specific relevance to phytochemical research. First, as an intact organism, zebrafish enable multi-target phenotypic screening that captures the polypharmacology inherent to many plant-derived compounds—effects that would be missed in target-centric biochemical assays (Knap et al., 2023). Second, zebrafish tolerate complex extracts such as decoctions and ethanolic preparations, preserving the potential synergistic interactions among constituents that are typically lost during early fractionation (Crawford et al., 2008). Third, the optical transparency of zebrafish larvae allows direct visualization of dynamic immune behaviors—including migration, phagocytosis, and tissue infiltration—offering a mechanism-agnostic, behavior-based readout particularly well suited to compounds with multi-target or undefined mechanisms (Meijer et al., 2014). Thus, while zebrafish represent a broadly powerful platform for anti-inflammatory drug discovery, their integrative and visual nature renders them especially well aligned with the chemical complexity and polypharmacology of plant-derived agents.

## 4 Multimodal integration and translational prospects of zebrafish-driven

**FIGURE 5:**
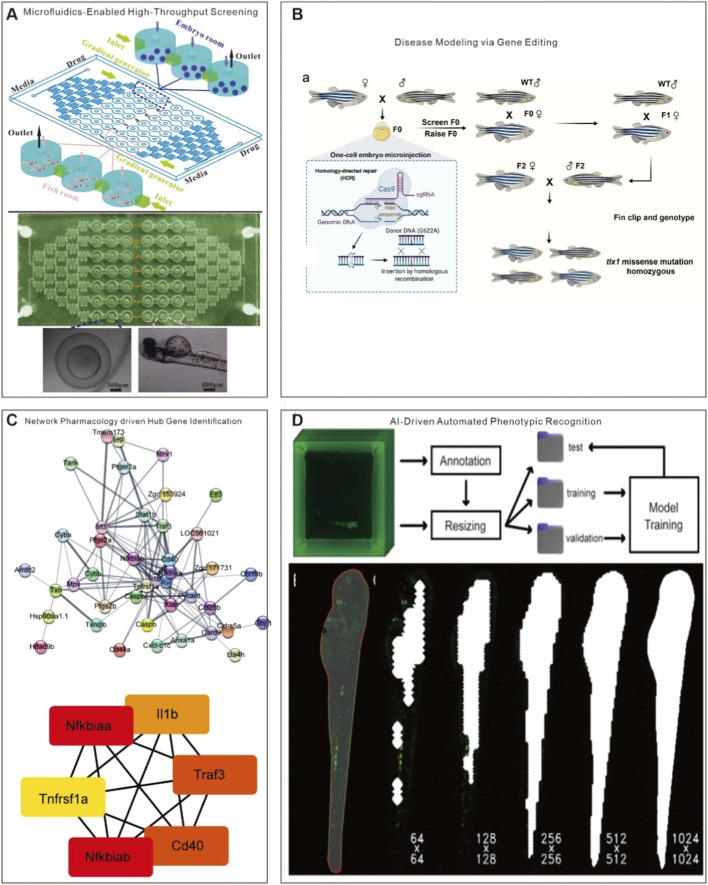
Applications of microfluidics, gene editing, network pharmacology, and artificial intelligence in zebrafish-based drug screening **(A)** Microfluidic chip platforms enable rapid and high-throughput drug screening. Reprinted with the permission from Ref. (Li et al., 2014). Copyright © 2014 PLOS **(B)** Gene editing technologies allow the generation of zebrafish models for specific disease contexts, facilitating pathophysiological simulation. Reprinted with the permission from Ref. (Liu et al., 2022). Copyright © 2022 Springer Nature **(C)** Network pharmacology facilitates efficient identification of key genes; the upper panel shows a protein–protein interaction (PPI) network constructed from differentially expressed genes, and the lower panel highlights the selected hub genes. Reprinted with the permission from Ref. (Tan et al., 2023). Copyright © 2023 Frontiers **(D)** AI–driven model training enables rapid and automated recognition of zebrafish phenotypes, streamlining the data analysis workflow. Reprinted with the permission from Ref. (Efromson et al., 2023).

Building upon the technology-augmented zebrafish framework outlined in Section 3.1, the platform has evolved from a validation tool into a multimodal discovery engine for anti-inflammatory phytomedicines. The zebrafish model—characterized by high-throughput screening capacity, optical transparency, genetic tractability, and cost efficiency—is rapidly transitioning from a basic research tool to a pivotal platform bridging mechanistic investigation and clinical translation (Figure 5A) (Patton et al., 2021; Yoganantharjah and Gibert, 2017; Li et al., 2014). Unlike conventional cellular and mammalian models, zebrafish offer distinct advantages for real-time live imaging, rapid genetic manipulation, and conserved immune and metabolic pathways—making them particularly suitable for mechanistic studies of natural products and novel compound screening (Sukardi et al., 2011). Gene-editing approaches (e.g., CRISPR–Cas9, TALEN) enable rapid generation of mutants (Figure 5B) (Liu et al., 2022). In another study, Efromson’s team established a high-throughput image analysis pipeline for fluorescent cells in zebrafish larvae. The integration of transcriptomics, metabolomics, and network pharmacology enables rapid and efficient screening of core differentially expressed genes from extracts (Figure 5C) (Tan et al., 2023). Using a *Tg(mpx:EGFP)* line, their system acquired images from a 96-well plate (∼18 GB) in 75 s and completed fluorescence-based cell counting in 5 min, demonstrating exceptional utility in large-scale genetic and compound screens (Figure 5D) (Efromson et al., 2023). Importantly, integration with emerging technologies such as microfluidics, AI, gene editing, network pharmacology, and humanized xenograft systems is transforming zebrafish from a validation model into a multimodal discovery platform for plant-derived anti-inflammatory agents.

### 4.1 Microfluidics-enabled high-throughput and high-content screening

Microfluidic technology allows precise fluid manipulation at the microscale, offering unparalleled advantages in evaluating natural product efficacy and optimizing delivery systems (Li et al., 2014). Its integration with zebrafish models facilitates large-scale drug screening with minimal sample consumption and enables systematic analysis of dose-response relationships and compound synergies through controlled concentration gradients and combinatorial strategies.

This integrated design addresses key limitations inherent to traditional high-throughput screening (HTS), namely, its low physiological relevance and inability to assess compound synergy in a controlled *in vivo* microenvironment, along with high compound consumption and reliance on static rather than real-time dynamic readouts. A representative application is the “Fish-in-Capsule” system developed by Tang et al. (2022). This droplet microfluidics-based approach immobilizes zebrafish larvae within agarose matrices, enabling automated, pipette-free drug exposure. When coupled with multispectral real-time imaging and machine learning algorithms, it allows acquisition and interpretation of high-dimensional phenotypic data. This integrated design addresses key limitations of traditional high-throughput screening (HTS), offering a robust platform for rapid validation and optimization of natural product combinations. Moreover, microfluidics has proven valuable in enhancing natural product delivery. For instance, baicalin-loaded liposomes (BCL-LPs) fabricated via this technology exhibited superior drug encapsulation and bioavailability in zebrafish, significantly augmenting anti-tumor and anti-inflammatory efficacy (Gu et al., 2024). Such approaches provide efficient delivery strategies for poorly soluble natural compounds and expand the therapeutic potential of nanocarrier systems in inflammation and oncology.

The Zebrafish Entrapment By Restriction Array (ZEBRA) device, introduced by Bischel et al., further enables high-throughput live imaging of zebrafish embryos (Bischel et al., 2013). This system allows rapid and predictable array-based positioning of embryos via simple pipetting, enhancing compatibility with automated microscopy. Using *Tg(mpx:dendra2)* zebrafish, the device validated leukotriene B4 (LTB4)-induced neutrophil migration to the tail fin and its inhibition by LY294002, demonstrating its utility for real-time analysis of immune cell dynamics (Xie et al., 2020).

The integration of microfluidics and zebrafish models represents not only improved experimental efficiency but also a conceptual shift—from testing single compounds to optimizing complex combination therapies. Future efforts should prioritize incorporating multi-channel compound exposure and high-content imaging within microfluidic platforms to identify synergistic dosing strategies of plant-derived anti-inflammatory compounds, thereby addressing the current lack of mechanistic and dosimetric rationale for clinical combination therapies.

### 4.2 AI-driven automated phenotypic recognition and data analytics

Manual phenotyping in zebrafish is labor-intensive, subjective, and unscalable: a single 96-well plate generates ∼18 GB of data requiring 6–8 h of manual analysis, with high inter-observer variability (Efromson et al., 2023). This throughput–resolution trade-off has long constrained phytochemical screening. Deep learning, originally developed for computer vision, overcomes this bottleneck by automating feature extraction, classification, and temporal tracking at speeds compatible with industrial-scale screening.

Owing to embryonic transparency, high-throughput capability, and genetic conservation of inflammatory pathways with humans, zebrafish are a powerful model for pharmacodynamic evaluation of natural products. However, conventional phenotype assessment relying on manual observation is subjective, low-throughput, and inadequate for large-scale screens. AI, particularly deep learning and machine vision, offers transformative potential to overcome this bottleneck (Wang N. et al., 2024).

Convolutional Neural Networks (CNNs) can automatically identify and classify key inflammatory phenotypes—such as neutrophil aggregation, vascularization, cardiac dynamics, and behavioral patterns—without human intervention. Recurrent Neural Networks (RNNs) and Transformer-based models can further capture temporal dynamics in developmental and inflammatory processes (Gutha et al., 2018; Barreiros et al., 2021; Lukovikov et al., 2024; Bozhko et al., 2022). For example, Thapa et al. developed a U-Net-based machine learning algorithm for automated identification and quantification of fluorescently labeled neutrophils in zebrafish, achieving an error rate of ≤8.65% and reducing processing time from hours to minutes (Thapa and Stachura, 2021). Notably, the pipeline is adaptable to various transgenic reporters and cell lineages, with the potential for to be applied across species. These advances illustrate how AI-powered phenotypic analysis provides a robust methodological foundation for rapid and precise screening of plant-derived anti-inflammatory compounds.

Looking forward, a promising direction involves developing algorithms that map zebrafish inflammatory scores to clinical biomarkers—such as C-reactive protein (CRP), erythrocyte sedimentation rate (ESR), or fecal calprotectin (Liu et al., 2020; Harrison, 2015; Sands, 2015). Integrating AI-based analysis of zebrafish behavioral and immune dynamics may enable the construction of quantifiable predictive models of clinical efficacy, addressing the translational gap between zebrafish data and clinical dosing. Although deep learning models have been successfully applied in toxicological assessments of zebrafish embryos (e.g., cardiotoxicity, neurobehavior) (Naderi et al., 2021; Dong et al., 2023), dedicated AI algorithms and standardized databases for phytochemical research remain scarce. With ongoing integration of computational biology, bioengineering, and multi-omics technologies, the zebrafish platform is poised to support a closed-loop translational pipeline—from *in vivo* screening and mechanistic investigation to clinical prediction.

A three-tiered iterative framework—combining graph neural network (GNN)-based molecular pre-screening, molecular dynamics simulations, and zebrafish live validation—could enable intelligent prediction of anti-inflammatory efficacy of plant-derived molecules. This paradigm may help unravel complex mechanisms of natural products and provide a predictive roadmap for personalized treatment of chronic inflammatory diseases. Future advancements in AI algorithms and cross-disciplinary technologies will likely position zebrafish as a core component of intelligent natural product screening platforms. By establishing open-access repositories for zebrafish imaging and omics data, and incorporating emerging approaches such as federated learning and explainable AI, we may achieve full automation from phenotypic recognition to mechanistic inference.

However, the integration of AI into zebrafish phytochemical research is hindered by three critical, yet frequently overlooked, barriers. First, the generalizability of algorithms remains largely unproven. Most deep learning models are trained on specific transgenic lines and imaging platforms, and consequently, they often fail to maintain performance when applied to divergent genetic backgrounds or disparate microscope hardware. Second, the field faces a bottleneck of annotation scarcity. Publicly available, expert-validated training datasets are virtually non-existent, compelling individual laboratories to generate costly labeled data. Third, current models lack biological explainability. Convolutional neural networks offer limited mechanistic insight; for instance, a classification of ‘responsive to treatment’ provides no elucidation regarding whether the observed effect is mediated by neutrophil apoptosis, reverse migration, or impaired endothelial adhesion. To overcome these limitations, future research must prioritize the development of open-source benchmark datasets, domain adaptation algorithms, and hybrid models that integrate imaging data with molecular readouts.

### 4.3 Synergistic integration of gene editing and network pharmacology in driving precision anti-inflammatory therapy and systems-level optimization

Contemporary strategies in anti-inflammatory drug development are transitioning from empirical approaches toward precision-targeted and system-level regulatory interventions. Gene editing technologies, particularly CRISPR activation systems, offer revolutionary tools for precise anti-inflammatory modulation by enabling targeted manipulation of key inflammatory regulatory nodes, such as the anti-inflammatory factors IL-10 and HO-1 (Cornet et al., 2018). For instance, specific activation of HO-1 gene expression has been shown to effectively suppress excessive release of TNF-α and IL-6, markedly ameliorating oxidative stress damage (Szumlak et al., 2024). Furthermore, the generation of transgenic zebrafish lines with immune cell-specific fluorescent markers allows real-time tracking of the effects of phytocompounds on neutrophils and macrophages *in vivo*, substantially enhancing the resolution of mechanistic drug studies (Ellett et al., 2011; Mao et al., 2025; Wang and Liu, 2020; Batista et al., 2018; Liu et al., 2024).

Nevertheless, inflammatory pathogenesis rarely hinges on a single gene and typically involves complex, multi-target networks and pathways. In this context, network pharmacology serves as a powerful complementary approach. By constructing comprehensive “compound–target–pathway–disease” networks and integrating multi-omics data, researchers can elucidate the polypharmacological mechanisms of natural products (Sachdev and Gupta, 2019; Chen X. et al., 2025). For example, combining pharmacodynamic assays, network pharmacology, and transcriptomics revealed the anti-inflammatory mechanism of Baoyuan Decoction in both CuSO_4_-induced and tail amputation-induced zebrafish inflammation modelsn (Zhou et al., 2023; Zheng et al., 2024). Similarly, network pharmacology uncovered the molecular basis by which the natural compound 2ʹ-hydroxychalcone ameliorates copper sulfate-induced inflammation in zebrafish (Shen et al., 2025), and clarified how *Moutan Cortex* (*Paeonia suffruticosa*) exerts its anti-inflammatory and blood-activating effects via modulation of the coagulation–inflammation cascade—a mechanism validated across cellular, rat, and zebrafish models. These cases underscore the utility of network pharmacology in decoding the pharmacodynamic material basis and holistic mechanisms of complex natural products.

Future research should foster deeper integration of gene editing and network pharmacology. This entails using network-based predictions to identify multi-target intervention points, followed by rapid genetic validation in zebrafish. Such a bidirectional cycle of *in silico* prediction and *in vivo* verification will considerably accelerate and improve the reliability of natural product research, transitioning from mechanistic hypothesis to mechanistic confirmation.

### 4.4 Humanized zebrafish models improve clinical translational relevance

The structural diversity and multi-target nature of phytochemicals make them promising candidates for anti-inflammatory drug development. However, their transition from basic research to clinical application remains fraught with unpredictability. While conventional zebrafish models offer advantages for high-throughput screening and mechanistic inquiry, physiological differences between zebrafish and humans limit their direct clinical relevance (Habjan et al., 2024). Recently, humanized zebrafish models—engineered to carry human cells, genes, or microbial communities—have significantly improved the physiological mimicry of human drug responses, offering enhanced biological relevance and translational value for preclinical evaluation of plant-derived anti-inflammatory compounds (Arjmand et al., 2020). When integrated with AI-driven phenotyping and multi-omics analyses, these models enable high-dimensional parsing of complex phenotypic data and cross-species mapping, strengthening the interpretability and clinical applicability of results (Balkrishna et al., 2020).

At the application level, transplantation of human immune cells into zebrafish enables modeling of human inflammatory responses, facilitating direct observation of how phytochemicals influence macrophage polarization, neutrophil migration, and inflammatory cytokine secretion. Incorporation of human hepatocytes or drug-metabolizing enzymes helps replicate human-specific metabolite profiles and potential toxicities. Moreover, transplantation of patient-derived tissues or organoids provides a platform for assessing individualized drug responses (Rajan et al., 2020; Häberlein et al., 2022). For example, grafting fragments of patient-derived pancreatic ductal adenocarcinoma into the yolk sac of wild-type AB zebrafish embryos has enabled the establishment of zebrafish patient-derived xenograft (zPDX) models (Yan et al., 2019a; Di Franco et al., 2020). Studies also show that in adult immunodeficient zebrafish (e.g., casper; *prkdc−/−*; *il2rga−/−*), human cells can achieve robust long-term engraftment (>28 days) (Yan et al., 2019b). A key advantage of adult models is the feasibility of clinically relevant drug administration routes, such as oral gavage or intraperitoneal injection. When combined with optimized transplantation sites (e.g., intraperitoneal, periocular, or intracranial) and advanced imaging modalities (e.g., confocal or two-photon microscopy), these models allow dynamic, single-cell-resolution monitoring of drug effects *in vivo*.

Using immunodeficient zebrafish strains and temperature control systems maintained at 37 °C, human cells—including immune, tumor, and stem cells—can be stably engrafted, survive, proliferate, and differentiate. These systems also support the transplantation of human peripheral blood mononuclear cells, macrophages, neutrophils, or iPSC-derived immune cells into immunocompromised or early embryonic zebrafish, facilitating the construction of inflammation models with a humanized immune background. Such models are invaluable for directly evaluating the effects of phytochemicals on human immune cell migration, activation, cytokine secretion, and polarization (M1/M2) (Somasagara et al., 2021; Verma M. et al., 2024). Notably, zPDX models have demonstrated promising correlations with clinical treatment responses in certain studies (Song et al., 2025; Tao et al., 2022; Fan et al., 2021). For instance, intestinal biopsy tissues from inflammatory bowel disease (IBD) patients transplanted into immunodeficient zebrafish have been used to validate the anti-inflammatory effects of plant compounds such as quercetin and chrysanthemum extract on patient-derived primary cells. Parallel pharmacodynamic comparisons with clinical drugs like 5-aminosalicylic acid (5-ASA) help establish translational links spanning *in vitro* screening, zebrafish validation, and clinical response (Li et al., 2022).

In summary, humanized zebrafish models—particularly using adult immunodeficient strains—have established mature protocols for transplantation, drug administration, and live imaging, providing a solid foundation for inflammatory disease research. The value of these models extends beyond verifying natural product efficacy; they also offer a scalable platform for personalized and precision medicine. Future studies could incorporate high-dimensional omics and AI analytics into zPDX platforms, forming an intelligent feedback loop of “clinical sample → zebrafish validation → efficacy prediction.” Although existing studies confirm that human cells can survive short-term and retain functionality in zebrafish embryos or larvae, and although transplantations of human tumor fragments or immune cells have yielded encouraging results in drug sensitivity and inflammation studies (Wang L. et al., 2021), challenges remain. These include limited long-term human cell survival due to temperature and physiological disparities, immune rejection, and a lack of standardized protocols and evaluation metrics. Continued advances in genetic engineering, temperature control, and humanization procedures are expected to gradually overcome these limitations.

The multimodal platform centered on zebrafish demonstrates substantial innovative and translational potential in anti-inflammatory phytodrug R&D. It incorporates microfluidic chip-based high-content screening, AI-powered phenotyping, CRISPR-mediated genetic manipulation, network pharmacology-guided polypharmacology, and xenograft-enhanced clinical modeling. Together, these technologies form a high-throughput, high-resolution, and highly predictive system for anti-inflammatory drug screening and mechanistic validation.

In conclusion, the zebrafish model is rapidly evolving from a basic research tool into an intelligent, systematic, and translation-ready platform for anti-inflammatory drug discovery. It is positioned to become a pivotal node connecting foundational biology and clinical application. The integration of AI, synthetic biology, multi-omics, and xenograft modeling will further refine its multimodal framework, ultimately yielding a closed-loop R&D pipeline encompassing deep mechanistic analysis, dynamic *in vivo* validation, and preclinical simulation. This evolution promises to significantly improve the clinical translation success of plant-derived anti-inflammatory drugs, transforming the discovery process from experience-dependent to mechanism-driven and systems-optimized. Ultimately, this will streamline the path from hit identification to clinical candidate development, offering novel strategies for the precise treatment of chronic inflammatory diseases (Table 4).

**TABLE 4:** Multimodal technology integration optimizing translational applications of zebrafish models in plant-derived anti-inflammatory research.

Technology module	Core advantage	Key application/Validation	Ref.
Microfluidics-driven HTS/HCS	• Precise fluid control^a^ • Automated embryo arraying • Integrated drug exposure	• *Fish-in-Capsule*: Automated zebrafish embedding + droplet microfluidics → synergistic anti-inflammatory compound screening • BCL-liposomes: 95.32% ± 0.48% encapsulation → enhanced anti-tumor efficacy and bioavailability in zebrafish vs. free baicalin • ZEBRA device: Rapid embryo positioning → validated neutrophil migration screening (LTB4-induced; LY294002-inhibited)	Tang et al. (2022), Gu et al. (2024), Bischel et al. (2013)
AI-powered screening	• Automated cell quantification (e.g., neutrophils)^b^ • High-speed image processing (e.g., 75 s/96-well plate) • Scalable framework for multi-lineage analysis	• ML algorithm: <8.65% error vs. manual counting; processing time reduced from hours to minutes • EGFP-neutrophil quantification: 18 GB image data analyzed in 5 min → high-throughput genetic/compound screening • Adaptable to diverse transgenic reporters	Thapa et al. (2021), Efromson et al. (2023)
Gene editing and network pharmacology	• CRISPRa-mediated^c^ • Transgenic reporters • Multi-target prediction	• HO-1 overexpression: Suppressed TNF-α/il-6 and oxidative stress in zebrafish • Network pharmacology: Identified sleep-regulating compounds from *Aquilaria* tea; predicted 2ʹ-Hydroxychalcone’s anti-inflammatory targets	Szumlak et al. (2024), Tan et al. (2023), Shen et al. (2025)
Humanized zebrafish models (e.g., zPDX)	• Humanized tumor/immune modeling in immunodeficient zebrafish^d^ • Long-term engraftment (>28 days in adults) • Clinical route simulation (e.g., oral gavage, IP injection)	• Real-time tracking of human rhabdomyosarcoma cell dynamics via photoconversion • zPDX (zebrafish patient-derived xenograft): Predictive of clinical drug response (e.g., olaparib + temozolomide) • Adult chimera: Enables high-resolution drug efficacy assessment at single-cell level	Habjan et al. (2024), Di Franco et al. (2020), Yan et al. (2019)

Abbreviations: HTS, High-Throughput Screening; HCS, High-Content Screening; LTB4, Leukotriene B4; zPDX, zebrafish Patient-Derived Xenograft.

^a^ Microfluidics: Requires specialized microfabrication facilities and expertise; chip design may limit throughput for certain applications; not all compounds are compatible with microfluidic channels due to solubility or adsorption issues.

^b^ AI-Powered Screening: Performance heavily depends on the quality and diversity of training datasets; risk of algorithmic bias; model predictions require experimental validation; interpretability of deep learning models remains challenging.

^c^ Gene Editing and Network Pharmacology: CRISPR-mediated editing may introduce off-target effects; network pharmacology predictions are correlative and require experimental confirmation; ethical considerations apply to germline editing.

^d^ Humanized Zebrafish Models: Immune reconstitution is often incomplete and variable between individuals; engraftment efficiency depends on donor cell quality; experimental windows are limited (typically 7–14 days in embryos, up to 28 days in adults); temperature maintenance at 32 °C–37 °C may affect zebrafish physiology.

While immunodeficient zebrafish models permit the engraftment of human immune cells, they possess an intrinsic limitation for studying immune-driven inflammatory processes that require functional adaptive immunity. The absence of endogenous T and B cells precludes the recapitulation of antigen-specific inflammation, memory responses, and lymphocyte-mediated chronicity in such chimeras (Barbosa et al., 2025). Moreover, the zebrafish microenvironment may lack key human cytokines and chemokines, potentially altering the behavior of engrafted human immune cells (Barbosa et al., 2025). Consequently, these models are best suited for investigating acute innate immune responses or tumor–immune interactions, whereas adaptive immune mechanisms are more appropriately studied in immunocompetent adult zebrafish or humanized mouse models.

Importantly, while the multimodal technologies summarized in Table 4 offer transformative potential for anti-inflammatory phytochemical research, they are not without inherent limitations. Each module carries specific caveats that must be carefully considered during study design and data interpretation. For instance, microfluidic systems demand specialized infrastructure; AI-driven screening is contingent on high-quality training data and may introduce algorithmic bias; gene editing techniques carry risks of off-target effects; and humanized zebrafish models exhibit incomplete immune reconstitution and variable engraftment efficiency (see Table 4 footnotes for detailed limitations). A balanced understanding of both the strengths and constraints of these approaches is essential to harness their full translational power and avoid overinterpretation of preclinical findings.

## 5 From model to clinic: translational integration, current challenges, and paradigm innovation of the zebrafish platform

Translating zebrafish findings to clinical applications requires navigating several inherent challenges, which are summarized below alongside strategic mitigation approaches. Utilizing classical inflammation models—such as those induced by CuSO_4_, lipopolysaccharide (LPS), or tail fin amputation—researchers can perform high-throughput efficacy screening. When combined with transgenic reporter lines and live imaging, the migration and functional dynamics of neutrophils and macrophages can be monitored in real time at single-cell resolution. The integration of microfluidic arrays and automated positioning systems (e.g., Fish-in-Capsule, ZEBRA) has substantially enhanced experimental scalability and imaging reproducibility. Furthermore, AI-driven image recognition and phenotypic scoring have improved the efficiency and objectivity of efficacy assessments. Meanwhile, the application of CRISPR-based gene editing and network pharmacology facilitates target validation and signaling pathway analysis of candidate molecules.

Through the continuous integration of multimodal technologies—including microfluidic chips, high-content imaging, AI, and genome editing—the zebrafish model not only supports an end-to-end pipeline from compound screening to toxicological evaluation but also demonstrates strong potential for translational applications encompassing target discovery, mechanism elucidation, and clinical correlation. For instance, studies have validated the utility of this platform in evaluating the efficacy and toxicity of plant-derived bioactive compounds such as xanthohumol and quercetin, underscoring its role as a critical bridge between basic research and clinical translation. By implementing a tiered screening strategy that incorporates *in silico* zebrafish modeling, high-throughput *in vivo* validation, and humanized/organoid reconfirmation, the zebrafish is increasingly recognized as a pivotal platform for the clinical translation of natural products. Coupled with federated learning and open data standards, cross-institutional data integration and model generalization can be achieved, thereby improving predictive accuracy and clinical relevance. This approach offers a viable pathway for personalized medicine and provides new scientific insights and translational opportunities for the safe and precise treatment of inflammatory diseases.

Despite its advantages, the zebrafish model faces inherent translational limitations that can be systematically categorized into four principal challenges, each addressable by emerging strategies. 1) Physiological disparities: as poikilotherms, zebrafish exhibit divergent drug metabolism and body temperature. Solution: CRISPR-engineered humanized metabolic strains (e.g., CYP3A4 knock-in) integrated with physiologically based pharmacokinetic (PBPK) modeling (Arjmand et al., 2020; Echeazarra et al., 2020; Wong et al., 2017). 2) Immature adaptive immunity: larvae lack key lymphocyte subsets, limiting chronic inflammation modeling. Solution: adult immunodeficient strains engrafted with human PBMCs or HSCs, enabling humanized immune chimeras (Yan et al., 2019a; Yan et al., 2019b). 3) Reductionist phenotyping: traditional readouts (e.g., neutrophil counts) fail to capture inflammation resolution dynamics. Solution: AI-driven multi-parametric imaging and spatiotemporal cell tracking (Knap et al., 2023; Green et al., 2024). 4) Incomplete tissue microenvironment: zebrafish organs differ in architecture and cellular complexity. Solution: co-transplantation of human organoids or patient-derived biopsies (zPDX) alongside phytochemical treatment (Song et al., 2025; Fan et al., 2021). Integrating these refinements will progressively close the translational gap, positioning zebrafish as a truly predictive preclinical platform for anti-inflammatory phytomedicines.

To mitigate metabolic and physiological disparities, “humanized” zebrafish models—such as those with knock-in of the human *CYP3A4* gene—can be generated via CRISPR/Cas9. Quantitative PK/PD models may then be developed by combining *in vivo* pharmacodynamic (PD) data from zebrafish with metabolism data from *in vitro* human liver microsomes, enabling more accurate dose extrapolation (Arjmand et al., 2020; Echeazarra et al., 2020; Wong et al., 2017). To enhance immune relevance, inflammation models in adult zebrafish—which possess a more developed adaptive immune system—should be further developed and utilized. These studies may be complemented by temperature control technologies (e.g., localized heating or the use of temperature-tolerant strains) to better approximate human physiological conditions (Renshaw and Trede, 2012; Traver et al., 2003; Garcia-Valtanen et al., 2017). A particularly promising strategy involves generating immunodeficient zebrafish (e.g., *casper*; *prkdc*−/−; *il2rga*−/−) engrafted with human PBMCs or HSCs to create zebrafish–human immune chimeras. Such models allow real-time visual tracking of human immune cells *in vivo*, substantially improving clinical translatability (Yan et al., 2019a).

**FIGURE 6:**
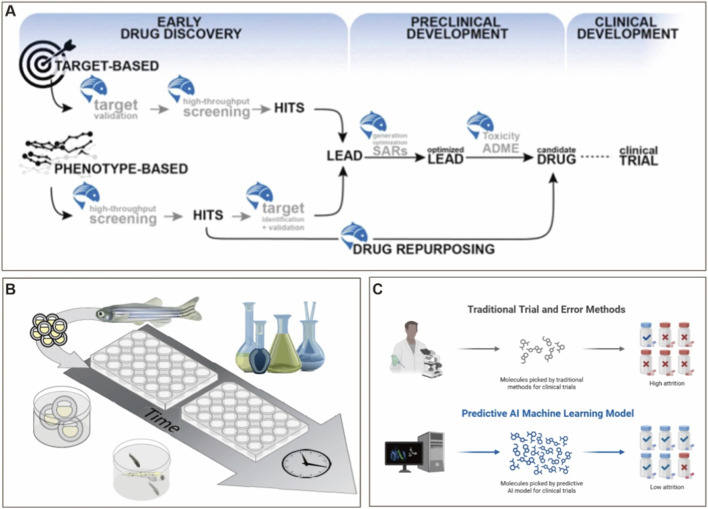
Applications of zebrafish in the discovery and development of plant-derived anti-inflammatory compounds **(A)** Schematic representation of the drug discovery pipeline using zebrafish, encompassing early-stage compound or natural product screening, preclinical evaluation, and clinical studies. Reprinted with the permission from Ref (185). Copyright © 2022 Frontiers **(B)** Illustration of high-throughput screening of natural extracts or compound libraries in the zebrafish model. Reprinted with the permission from Ref (186). Copyright © 2024 Frontiers **(C)** Comparative illustration of screening efficiency between conventional drug discovery approaches and AI-driven machine learning strategies. This figure was created with BioRender (https://biorender.com).

Application of deep learning and multimodal imaging can overcome the constraints of traditional quantification methods (Figure 6A) (Ochenkowska et al., 2022). These approaches enable systematic analysis of immune cell dynamics, cell–cell interactions, and pathologic microenvironments within tissues, leading to a more comprehensive understanding of inflammatory phenotypes (Green et al., 2024). In summary, advancing the clinical translation of the zebrafish platform for plant-based anti-inflammatory research depends on establishing a closed-loop framework centered on “multimodal data integration–AI-driven prediction–humanized model validation” (Knap et al., 2023). By deeply integrating high-content zebrafish phenotypic data with multi-omics profiles (e.g., transcriptomics, proteomics, and metabolomics), and leveraging graph neural networks with multi-task learning algorithms, a virtual zebrafish efficacy prediction platform can be built (Figure 6B) (Garg and Geurten, 2024). This system would enable *in silico* pre-screening of natural products, with top candidate compounds subsequently validated in human organoid–zebrafish chimera models. Such a strategy promises to markedly improve the precision and efficiency of anti-inflammatory drug discovery (Figure 6C). The zebrafish is evolving from a basic model into an intelligent, translational drug discovery engine, accelerating plant-derived anti-inflammatory applications and enabling precision treatment of inflammatory diseases.

## 6 Conclusion

Zebrafish-model provides an integrated pipeline spanning initial *in vivo* screening, toxicity evaluation, and mechanistic investigation, thereby serving as a pivotal translational bridge in the discovery and development of phytochemical-based anti-inflammatory therapeutics. This review systematically summarizes recent advances in the application of zebrafish models to study plant-derived anti-inflammatory compounds, encompassing widely used inflammation models, live imaging and transgenic lineage tracing, high-throughput, high-content screening strategies, and mechanistic insights. Furthermore, we discuss the integration of zebrafish models with multidisciplinary approaches, including network pharmacology, gene editing, AI-driven phenotype recognition, and humanized model. This integrated strategy is anticipated to open new avenues for personalized anti-inflammatory drug development, providing robust scientific and translational support for the safe and precise treatment of inflammatory diseases.

## Glossary

NF-κB: Nuclear Factor Kappa-Light-Chain-Enhancer of Activated B Cells
MAPK: Mitogen-Activated Protein Kinase
Nrf2: Nuclear Factor Erythroid 2-Related Factor 2
LPS: Lipopolysaccharide
COX-2: Cyclooxygenase-2
5-LOX: 5-Lipoxygenase
NLRP3: NLR Family Pyrin Domain Containing 3
AI: Artificial Intelligence
CRISPR/Cas: Clustered Regularly Interspaced Short Palindromic Repeats/CRISPR-Associated Protein 9
TALEN: Transcription Activator-Like Effector Nuclease
DSS: Dextran Sulfate Sodium
IL: Interleukin
TNF-α: Tumor Necrosis Factor-Alpha
FA: Forsythiaside A
FB: Forsythiaside B
ROS: Reactive Oxygen Species
MDA: Malondialdehyde
MPO: Myeloperoxidase
SOD: Superoxide Dismutase
GSH-Px: Glutathione Peroxidase
iNOS: Inducible Nitric Oxide Synthase
qRT-PCR: Quantitative Real-Time Polymerase Chain Reaction
HTS: High-Throughput Screening
HCS: High-Content Screening
PDX: Patient-Derived Xenograft
zPDX: zebrafish Patient-Derived Xenograft
TREM2: triggering receptor expressed on myeloid cells 2
IBD: Inflammatory Bowel Disease
PK/PD: Pharmacokinetic/Pharmacodynamic
ADME: Absorption, Distribution, Metabolism, Excretion
CYP3A4: Cytochrome P450 Family 3 Subfamily A Member 4
PBMC: Peripheral Blood Mononuclear Cells
HSCs: Hematopoietic Stem Cells
iPSCs: Induced Pluripotent Stem Cells
QFAE: Quzhou Fructus *Aurantii* Extract
LRE: *Lycium ruthenicum* ethanol extract
EO: Essential Oil
BJL: Tribulus terrestris extract
ECGC: Epigallocatechin Gallate
STD: *Stytontriterpene* D
TH: Tuberoside H
ZEBRA: Zebrafish entrapment by restriction array
CNN: Convolutional Neural Network
RNN: Recurrent Neural Network
GNN: Graph Neural Network
LTB4: Leukotriene B4
CRP: C-Reactive Protein
ESR: Erythrocyte Sedimentation Rate
5-ASA: 5-Aminosalicylic Acid.
